# Use of statins in lower extremity artery disease: a review

**DOI:** 10.1186/1471-2482-12-S1-S15

**Published:** 2012-11-15

**Authors:** Giuseppe Gargiulo, Giuseppe Giugliano, Linda Brevetti, Anna Sannino, Gabriele Giacomo Schiattarella, Federica Serino, Andreina Carbone, Fernando Scudiero, Marco Ferrone, Roberto Corrado, Raffaele Izzo, Lorenzo Chiariotti, Cinzia Perrino, Bruno Amato, Bruno Trimarco, Giovanni Esposito

**Affiliations:** 1 Department of Clinical Medicine and Cardiovascular and Immunology Sciences, Federico II University, via Pansini 5, 80131, Naples, Italy; 2 Department of Biology and Cellular and Molecular Pathology, Federico II University, via Pansini 5, 80131, Naples, Italy; 3 Department of General, Geriatric, Oncologic Surgery and Advanced Technologies, Federico II University, via Pansini 5, 80131, Naples, Italy

## Abstract

**Background:**

Lower extremity artery disease (LE-PAD) is one of the most common manifestations of atherosclerosis, particularly in elderly patients, and it is related to a high cardiovascular risk.

**Description:**

It is well established that statin therapy is characterized by crucial benefits on cardiovascular system by limiting atherosclerotic progression and reducing cardiovascular events and mortality. A growing body of evidence support efficacy of statins in LE-PAD due to the ability of both reducing cardiovascular risk and improving walking distance and, hence, quality of life. Consequently, statin therapy should be considered in all LE-PAD patients and new LDL-cholesterol targets should be reached.

**Conclusions:**

Our opinion is that statin therapy remains still underutilized or with inadequate dosage, so therapy of LE-PAD patients should be improved to obtain all the demonstrated benefits of statins.

## Background

Lower extremity artery disease (LE-PAD) is one of the most common manifestations of atherosclerosis and its frequency is strongly related to age: uncommon before 50 years, rising steeply at older ages. A substantial percentage of patients with chronic coronary artery disease (CAD) have associated cerebrovascular disease, LE-PAD, or both. Consequently, LE-PAD represents a marker of diffuse atherosclerosis implying a high cardiovascular risk [1-3] and, in symptomatic patients, it is also an important cause of disability. Secondary prevention of cardiovascular risk factors is mandatory in all LE-PAD patients to improve cardiovascular prognosis, while revascularization should be restricted to symptomatic patients. In order to improve symptoms and walking distance capacity conservative or invasive approaches (endovascular or surgical revascularization) can be undertaken. The conservative strategy is effective and based on pharmacologic agents (anti-platelet, lipid-lowering, antihypertensive; cilostazol; naftidrofuryl; pentoxifylline; carnitine; buflomedil) and exercise therapy, whose beneficial effects on LE-PAD and the cardiovascular system are well established [1,4]. While revascularization is recommended in patients with critical limb ischemia (CLI), the evidence of any long-term benefit of endovascular treatment over supervised exercise and best medical treatment is inconclusive, particularly in patients with mild to moderate claudication. However, advances in the endovascular treatment of LE-PAD have prompted many physicians to consider more liberal indications for percutaneous intervention. Endovascular revascularization is also indicated in patients with lifestyle-limiting claudication when clinical features suggest a reasonable likelihood of symptomatic improvement and there has been an inadequate response to conservative therapy. In aorto-iliac lesions, endovascular revascularization can be considered without initial extensive conservative treatment. Endovascular revascularization for the treatment of patients with LE-PAD has developed rapidly during the past decade, and a great number of patients can now be offered the less invasive treatment option. Despite numerous advantages, the major drawback of endovascular interventions, compared with surgery, is the lower long-term patency, mainly due to restenosis [5-9]. However, an increasing number of centres favour an endovascular approach first, due to reduced morbidity and mortality, compared with vascular surgery, while preserving the surgical option in case of failure.

## Methods

Pleiotropic effects and cardiovascular benefits of statin therapy are well-established [10-14], in particular their beneficial effects on atherosclerosis (reduction of cholesterol levels, inhibition of inflammation and plaque stabilization). In addition, statins reduce the risk of mortality, cardiovascular events, and stroke in patients with LE-PAD with and without coronary artery disease (CAD) [10,11].

According to these considerations, LE-PAD treatment has two main objectives: 1) to reduce cardiovascular risk, 2) to improve walking distance and, hence, quality of life. A large body of evidence demonstrates that statins exert positive effects on both. In a retrospective trial, Aronow and Ahn [15] observed a significant reduction of coronary events in 318 LE-PAD patients treated with statins related to 342 untreated patients. Shillinger et al. [16], in a prospective non-randomized trial, described that LE-PAD patients treated with statins had a halved risk of death and myocardial infarction. This latter result was considered predominantly due to the anti-inflammatory effects of statins, since patients with low levels of C-Reactive Protein (CRP) did not evidence a significant benefit from this therapy. Feringa et al. [17], in a 8-years perspective trial, demonstrated that the use of statins was associated to a reduced incidence of death (HR = 0,46, 95% CI 0,58-0,80, p<0.001). In the REGRESS trial [18], designed in order to test the effects of two-year-treatment with pravastatin on coronary arteries, it was demonstrated also a significant reduction of carotid and femoral intima-media thickness (assessed by echo-color-doppler) and a significant reduction of cardiovascular events compared to placebo. In the Heart Protection Study [10], a double blind randomized study, 6748 participants had PAD; at 5-year-follow-up, simvastatin-treated patients showed a significant 19% relative reduction and a 6.3% absolute reduction in major cardiovascular events related to placebo-treated patients, independently of age, gender, or serum lipid levels.

Beyond the evidence that statins improve the cardiovascular prognosis of patients with LE-PAD, several studies reported preliminary positive effects of statins on intermittent claudication [19,20]. The increase in maximal walking distance reported varied, on average, from 50 to 100 m. In one meta-analysis, the pooled effect estimate showed a relevant increase in maximal walking distance of 163 m^16^. In the 4S trial [21], simvastatin reduced incidence or worsening of claudication in hypercholesterolemic patients. McDermott et al. [22] described a better walking performance in LE-PAD patients treated with statins, although these benefits were attenuated when considered CRP levels. The beneficial effects of simvastatin on walking distance in LE-PAD patients were also shown by Aronow et al. and Mondillo et al. [23]. Mohleret al. [24] demonstrated that atorvastatin prolongs walking distance without claudication and improves quality of life. Based on this growing evidence, recent European and American guidelines consider LE-PAD at the same level of CAD, recommending the use of statins in all LE-PAD patients in the absence of contraindication. Particularly, serum LDL cholesterol should be reduced to <2.5 mmol/L (<100 mg/dL), and optimally to <1.8 mmol/L (<70 mg/dL), or ≥50% LDL cholesterol reduction when the target level cannot be reached [1].

## Conclusions

Our opinion is that statin therapy remains still underutilized or with inadequate dosage, so therapy of LE-PAD patients should be improved to obtain all the demonstrated benefits of statins.

## List of abbreviations

CAD: Coronary artery disease; CLI: Critical limb ischemia; CRP: C-Reactive Protein; LDL: Low density lipoprotein; LE-PAD: Lower extremity artery disease.

## Competing interests

The authors declare that they have no competing interests.

## Authors’ contributions

GG, GG: conception and design, drafting the manuscript, given final approval of the version to be published; LB, AS, GGS, FS, AC, FS, MF, RC, RI, LC: acquisition of data, drafting the manuscript; CP, BA, BT: critical revision, given final approval of the version to be published; GE: conception and design, critical revision, given final approval of the version to be published.
